# PRIMA subretinal wireless photovoltaic microchip implantation in non-human primate and feline models

**DOI:** 10.1371/journal.pone.0230713

**Published:** 2020-04-08

**Authors:** Mahiul M. K. Muqit, Jean Pierre Hubschman, Serge Picaud, Douglas B. McCreery, Jan C. van Meurs, Ralf Hornig, Guillaume Buc, Martin Deterre, Céline Nouvel-Jaillard, Elodie Bouillet, Claire-Maelle Fovet, Philippe Hantraye, José Sahel, Joseph N. Martel, Yannick Le Mer

**Affiliations:** 1 Vitreoretinal Service, Moorfields Eye Hospital, London, United Kingdom; 2 Institute of Ophthalmology, University College London, London, United Kingdom; 3 Stein Eye Institute, University of California Los Angeles, Los Angeles, CA United States of America; 4 Sorbonne Université, INSERM, CNRS, Institut de la Vision, Paris, France; 5 Huntington Medical Research Institutes, Pasadena, CA, United States of America; 6 Rotterdam Eye Hospital, Rotterdam, The Netherlands; 7 ErasmusMC, Rotterdam, The Netherlands; 8 Pixium Vision, Paris, France; 9 Molecular Imaging Research Center (MIRCen), CEA, Fontenay aux Roses, France; 10 Hopital des Quinze Vingts, Paris, France; 11 Retina and Vitreous Service, University of Pittsburgh Medical School, Pittsburgh, PA, United States of America; 12 Fondation Ophtalmologique A. De Rothschild, Paris, France; Massachusetts Eye & Ear Infirmary, Harvard Medical School, UNITED STATES

## Abstract

**Purpose:**

To evaluate the surgical technique for subretinal implantation of two sizes of PRIMA photovoltaic wireless microchip in two animal models, and refine these surgical procedures for human trials.

**Methods:**

Cats and Macaca fascicularis primates with healthy retina underwent vitrectomy surgery and were implanted with subretinal wireless photovoltaic microchip at the macula/central retina. The 1.5mm PRIMA chip was initially studied in feline eyes. PRIMA implant (2mm,1.5mm sizes) arrays were studied in primates. Feasibility of subretinal chip implantation was evaluated with a newly-developed surgical technique, with surgical complications and adverse events recorded.

**Results:**

The 1.5mm implant was placed in the central retina of 11 feline eyes, with implantation duration 43–106 days. The 1.5mm implant was correctly positioned into central macula of 11 primate eyes, with follow-up periods of minimum 6 weeks (n = 11), 2 years (n = 2), and one eye for 3 years. One primate eye underwent multi-chip 1.5mm implantation using two 1.5mm chips. The 2mm implant was delivered to 4 primate eyes. Optical coherence tomography confirmed correct surgical placement of photovoltaic arrays in the subretinal space in all 26 eyes. Intraoperative complications in primate eyes included retinal tear, macular hole, retinal detachment, and vitreous hemorrhage that resolved spontaneously. Postoperatively, there was no case of significant ocular inflammation in the 1.5mm implant group.

**Conclusions:**

We report subretinal implantation of 1.5mm and 2mm photovoltaic arrays in the central retina of feline and central macula of primate eyes with a low rate of device-related complications. The in vivo PRIMA implantation technique has been developed and refined for use for a 2mm PRIMA implant in ongoing human trials.

## Introduction

**Fig 1:**
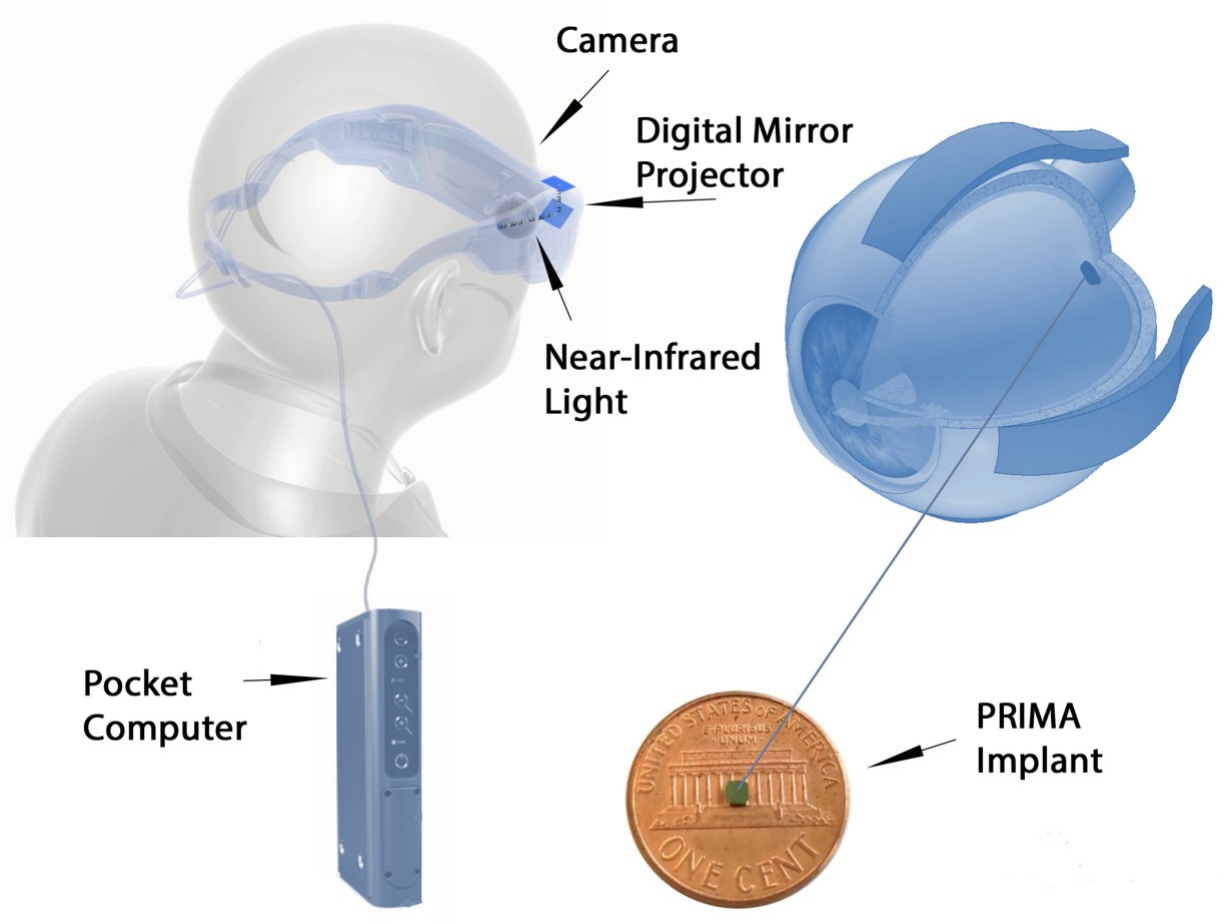
Schematic of PRIMA system.

The PRIMA implant is a wireless photovoltaic microchip array for subretinal stimulation [1]. In this optoelectronic prosthetic system, each pixel of the subretinal array photovoltaically converts patterned pulsed near-infrared (NIR) light projected from video glasses into pulses of bi-phasic electric current to stimulate the inner retinal cells in front of it [2–4]. The interface with the visual environment is achieved through a mini camera integrated in a pair of glasses that captures the overall field of view ("visual scene"). The visual information is then processed and converted to stimulation information which is used to activate the implanted retinal prostheses ("Implants"). Stimulation waveforms of infrared light are projected into the eye, through a near-to-eye digital mirror projection system. When the gaze direction is such that some part of the implant is illuminated by part of the stimulation data, the photovoltaic retinal implant converts that part of the signal into electrical current that stimulates the retina accordingly (Fig 1).

Different types of photovoltaic arrays have been studied in rat and pig eye models by Adekunle and coworkers, and the biocompatibility and subretinal integration did not show any significant adverse issues [5]. Lorach and Palanker’s group have clearly demonstrated the direct electrical relationship and interaction between natural visually evoked signals and prosthetic responses in a photovoltaic subretinal implant in Long Evans rat models [6]. Improvements in visual acuity have been demonstrated in blind animal models compared to healthy rat models [7]. In 2018, Ho and co-workers compared prosthetic stimulation in blind retinal degenerate Royal College of Surgeon rats implanted with subretinal 1mm and 2mm photovoltaic arrays compared to healthy Long Evan rats [8]. Near infrared light stimulated the blind animals with startling responses noted in behaviour patterns at pulsed electrical thresholds.

The safety of a similar implant has been shown in a human clinical trial [9]. The artificial silicon retina (ASR) microchip (Optobionics Corp, Naperville, Illinois, USA) was a 2mm diameter semi-conductor microphotodiode array of 25μm thickness designed for subretinal implantation. In a pilot 18-month study, the ASR was safely implanted in patients with retinitis pigmentosa with no significant adverse chip-related or surgical complications, and improved visual function was reported [9]. The biocompatibility, electrical safety and targeted location of the ASR within the subretinal space have been reported in animal studies, with optimal stimulation resolution from the subretinal ASR [10–12].

The translation of this photovoltaic retinal implant evaluation to human clinical trials with defined surgical protocols and assessment of functional outcomes is the next step. Our initial study aimed at evaluating the feasibility of subretinal 1.5mm PRIMA chip implantation in a feline model. Two different sizes of PRIMA implants were then studied in non-human primate retinal model. The goal of these in vivo studies were to further refine surgery protocols, and to evaluate complications, chip integrity, and chip stability when placed in the subretinal space before translation to human trial.

## Materials and methods

### Study design

The feline study was conducted in 2015. Surgical procedures and follow-up were performed at Huntington Medical Research Institutes (HMRI) who are accredited by the American Association for Laboratory Animal Care (AALAC). All animals were followed-up throughout the study by a veterinary physician. The study was approved by the institutional review board of HMRI (Protocol Number: R15017). The animals were managed according to standard internal policy on animal welfare and care at HMRI. The studies followed the WMA statement on animal use in biomedical research.

The sequential non-human primate animal study was conducted between 2015 and 2018. Surgical procedures and follow-up were performed at the Molecular Imaging Research Center (MIRCen- CEA, Fontenay aux Roses), in cooperation with Institut de la Vision, Paris, and institutional review board approval was in place. The study was approved by the institutional review board of Institut de la Vision and MIRCen-CEA (Protocol Number: R16014; R16025; R16050; and, R16064;). The animals were managed according to standard internal policy on animal welfare and care at MIRCEN. The studies followed the WMA statement on animal use in biomedical research.

The primary aims of the study were to establish an implantation procedure of two sizes of PRIMA implant in feline and non-human primate models. The secondary aims were to assess implant delivery at the appropriate location at the macula, with the electrode side up, and without implant debris left in the eye. We aimed to assess for surgical complications related to the implant and to the surgery procedure itself including significant inflammations, retinal damage, choroidal damage, and/or other adverse effects on the eye tissues. Adverse events (AE) and serious adverse events (SAE) were defined by standard BS EN ISO 14155:2011.

### Animal models

#### Feline

The cats were housed in Huntington Medical Research Institutes (HMRI). The HMRI follows national standards for animal welfare according to ARRIVE guidelines. The Animal Care and Use program of HMRI is accredited by the Association for Advancement and Accreditation of Laboratory Animal Care (AAALAC). Facilities and records are inspected regularly by a representative from the United States Department of Agriculture. All animal studies were performed in accordance with the standards set forth in the Guide for the Care and Use of Laboratory Animals (8^th^ edition) and are approved by the HMRI Animal Care and Use Committee. All cats were purchased from Liberty Research. All cats received fresh water and food daily according to their nutritional needs. They were fed a standard Feline Laboratory diet. They resided in the outdoor group housing exercise areas on most days and were adapted to outdoor temperatures through daily exposure, except in inclement weather. A daily exercise log was maintained for the outdoors exercise area, including the minimum and maximum daily temperature. Aseptic surgical procedures, including implantation of the PRIMA device were performed in a dedicated surgical suite. After the surgery the cat was visually monitored and vital signs recorded every 15 minutes until the animal was able to maintain sternal recumbency, at which time they received an injection of bupronorphine for pain relief. When recovered from anesthesia the cats were returned to the primary animal care facility. During the first 4 days after the implant surgery they were housed in a large individual cage (16 ft^2^) and were not released into the outside exercise area so that they may be observed more closely. At night the cats were housed and fed in their individual cages, each of which is 4 ft^2^, with an elevated perch. To encourage resumption of eating after the implant surgery they are offered moist commercial cat food in addition to the standard feline laboratory diet. All animals were subject to an eye examination by the Study Veterinary Physician after 1 day then 8 days post-operatively. Fundus photography and OCT images were performed after surgery. Slitlamp examination of the anterior chamber was performed in order to detect any sign of inflammation, and eye pressure was recorded. At the end of this study, the disposition of the animals was by euthanasia. Animals were sacrificed by overdose of pentobarbital.

The feline model was selected for this study because the feline retina approximates the human retina. In both cat and human, the retina contains rods and cones and have a central region of high cone density. The cats used for this study were healthy, domestic, males with normal vision and aged between eleven [11] and sixteen [16] months at the time of intervention. A 1.5mm implant was delivered into 11 eyes, and follow up was between 43 and 106 days. All surgeries were performed under general anesthesia.

#### Non-human primate

The animal model was the Macaca fascicularis and MIRCen-CEA follows national standards for animal welfare according to ARRIVE guidelines.

All animal experimentations were performed by experienced veterinary surgeons, in conformity with national and European laws. The non-human primate species macaca fascicularis (common name cynomolgus monkey), male or female, originate from a breeder in Mauritius. The animal facility is authorized for animal experimentation on non-human primates (n° D 92 032 02) by local authorities and complies with the EU directive requirements regarding the use of animal in research (2010/63/EU). Animals are subjected to daily controls, which include clinical and behavioural assessments. Trained staff was responsible for care and housing procedures (animal caretakers, veterinarians and neuroscientists). Housing facility is equipped with an HVAC (heating, ventilation and air conditioning) secured system. Animals are housed socially in caging systems that allow vertical movement and space to rest. The cage’s orientation within the room allows the animals to see each other and to maintain social contact (facial mimics and vocalizations). All cages are equipped with a squeeze back to restrain the animal gently and to minimize the physical contact. Animals are housed in groups (minimum pair) when the scientific goals of the study allow it. If not, the isolated animal is given specific environmental enrichments and the visual contact with a social partner is maintained. Positive reinforcement is used in the everyday procedures.

Animals received fresh water and food daily according to their nutritional needs. Extruded pellets are specifically manufactured to fulfil macaque’s nutritional needs (Altromin, ND). Animals are provided with environmental enrichment to express their natural behaviours that minimize environmental change and enhance their homeostasis. The environmental enrichment program is standardized and includes food enrichment (daily fruits, weekly access to seeds boxes/bags) and manipulating tools (mirrors, triangles, dental toys). Smooth music is played during working hours to cover unexpected sounds and to provide an acoustic enrichment. Lights turn on and off under a progressive program that mimics dawn and sunset.

General anesthesia was used for all surgery and longitudinal examinations. The induction of anaesthesia used intramuscular ketamine 10mg/kg and xylazine 0.5mg/kg after fasting the day before the surgery. Maintenance of anesthesia used intravenous propofol 1ml/kg/h with oxybuprocaine for local anesthesia of the eye. Intubation of the animal was performed as a precautionary measure during surgery

Humane endpoints were followed with non-human primates only receiving an implant in one eye. Procedure specific humane endpoints were implemented in case of problems as indicated below. In order to quantify the impact of experimental procedures on the animals, we proposed a graded classification of ocular problem severity that included blindness, conjunctivitis, orbital swelling, and cataract. Nonspecific Humane endpoints were applied according to the “Humane Endpoint Guidelines for Nonhuman Primates in Biomedical Research” (Association of Primate Veterinarians). Specific humane endpoints for this procedure included: Binocular blindness grade 2 for more than 15 days; glaucoma grade 2 non-responsive to treatment (15 days); conjunctivitis grade 2 non-responsive to treatment (15 days); non-specific pain symptoms; and, anorexia for 3 days/ lost weight above 15% of initial weight. Special care was given to avoid rank fights that might arise from impaired vision in one eye. non-human primates having received implant were observed closely (each day after surgery) for the procedure specific impact. If social interactions between non-human primates undergoing the study changed, they were housed in separate cages while ensuring social interaction across the cages (animals were still able to see each other).

Close-monitoring of the animals was conducted, and follow-up was conducted in the 1.5mm implantation group. Fundus photography and OCT images were performed: 1 day, 2, 4, 6 weeks after surgery; and, at 1–3 years in some eyes after surgery. Slitlamp examination of the anterior chamber was performed in order to detect any sign of inflammation, and eye pressure was recorded. If required, the disposition of the non-human primate was through euthanasia. If euthanasia was performed, animals were initially calmed in their cages by intramuscular injection of a mixture of Ketamine at 10 mg/kg and xylazine (0.5mg/kg). A barbiturate was then injected intravenously (pentobarbital at 180mg/kg), and the eyes were enucleated.

**Fig 2:**
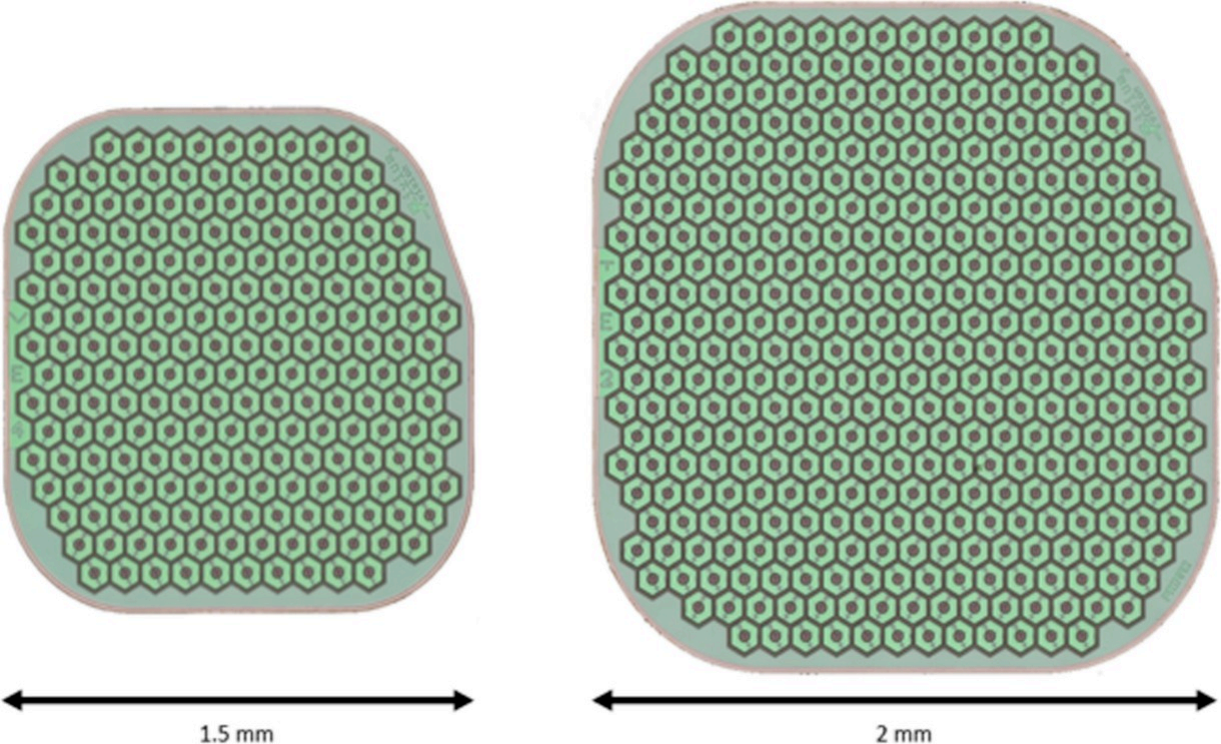
Design of PRIMA retinal implants.

The diameter of the eye in this species is approximately 18 to 20mm. Anatomy is very close to human, and the retina and eye examination was normal in each eye studied. All surgeries were performed in sterile conditions under general anesthesia by veterinary anesthesiologists. The 1.5mm implant (Fig 2) was delivered in total 11 eyes.

In one animal, the feasibility of the surgical procedure to deliver multi-chip implantation of two 1.5mm arrays was evaluated. The follow-up period was 4 weeks (n = 11) 2 years (n = 2), and 3 years (n = 1) for the 1.5mm implanted animals. The 2mm implant (Fig 2) was delivered to 4 eyes of four animals. All animals implanted with a 2mm implant were euthanized after the intervention.

### Surgery procedure

**Fig 3:**
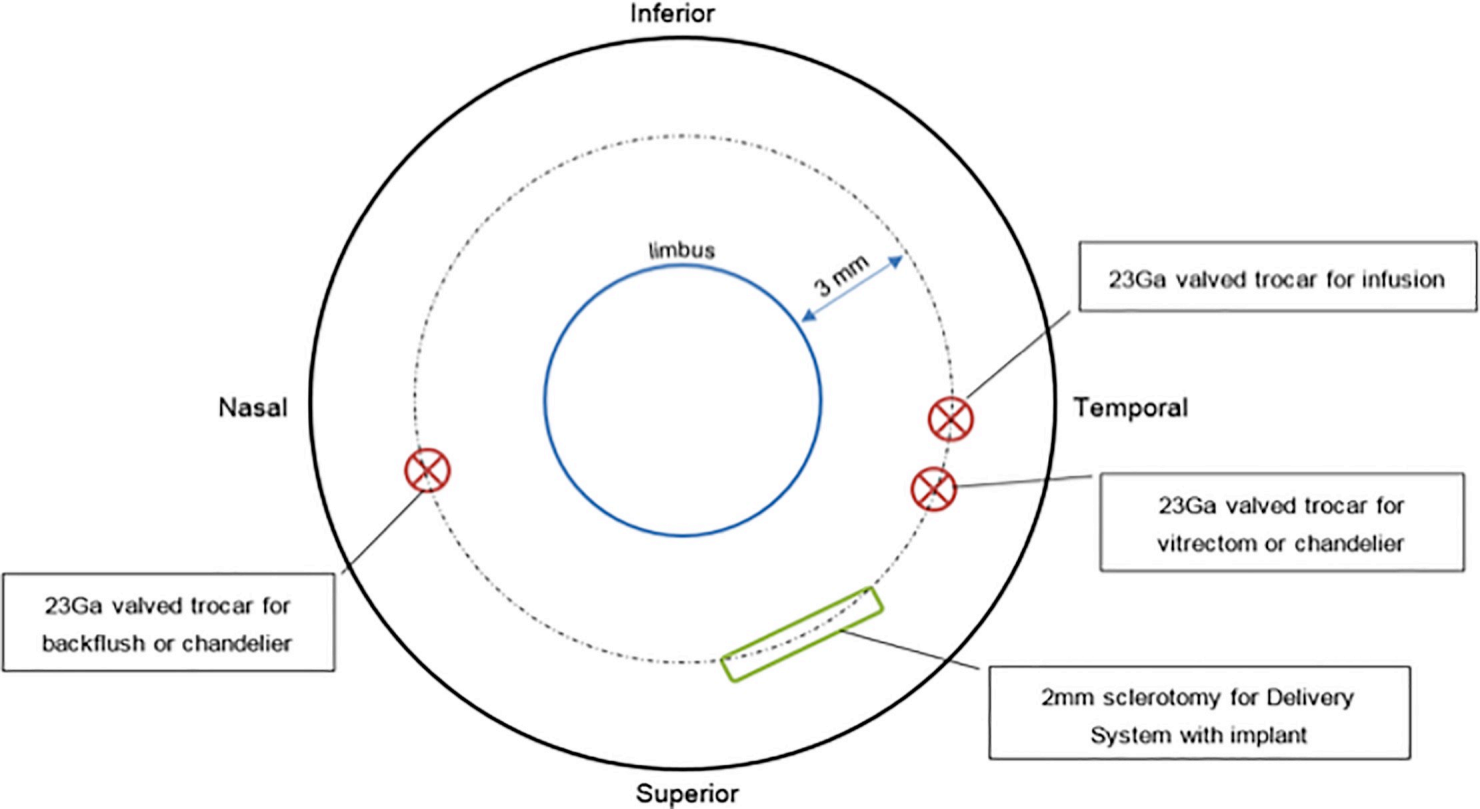
Diagram of vitrectomy instrumentation entry ports.

The PRIMA surgery was performed according to a defined surgical protocol by the authors (YlM, JH) who both underwent a standardized briefing and training with the original surgical protocols for the studies. In this way, the different steps of the surgical technique could be assessed in different surgical conditions/models and using two independent operators. There is a time lag between studies due to the time for different country regulatory approvals to be completed. The surgery technique was identical in both the feline and primate studies and is detailed here in different steps:

Step 1: Three 23-gauge (23G) sclerotomies were placed 3mm posterior to the limbus, and standard pars plana vitrectomy (PPV) was performed using a Synergetics Versavit (Synergetics USA Inc) for the cat study, and Alcon Constellation (Alcon Laboratories Inc, Fort Worth, TX) for the primate study. The Resight 500 wide-angle viewing system and Zeiss Lumera 700 microscope (Carl Zeiss Meditec AG, Jena, Germany) was used for primate surgery under general anesthesia (Fig 3).
Step 2: Triamcinolone acetonide (Kenalog-40, Bristol Myers Squibb, Princeton, NJ) was injected at the beginning of the vitrectomy through the 23G trocar to ensure complete posterior vitreous detachment and vitrectomy.
Step 3: A subretinal injection of balanced salt solution was delivered to create a bleb in the temporal part of the posterior pole using a 41-gauge subretinal cannula. Several injections could be used to enable detachment of the central macula (primate) /central retina (feline).
Step 4: A 3mm area of the temporal macula, near or between the upper retinal vessels was coagulated with endodiathermy. This coagulated area marks the entry point of the implant, and was aligned parallel to the sclerotomy.
Step 5: A retinotomy was made with vertical subretinal scissors, at the superior temporal part of the subretinal fluid bleb. The size of the retinotomy was calculated using a scaling method of preoperative optic nerve imaging. On average, the length of the retinotomy was 2 times the longest dimension of the optic nerve. The size of this retinotomy was enlarged for the 2mm implant insertion compared to the 1.5mm implant.
Step 6: A new 2.4mm sclerotomy was created 3mm from the limbus, and the choroid cauterized behind the incision. This would minimize potential intraocular hemorrhage during implant insertion. Sutures of 6/0 vicryl were preplaced at the large sclerotomy site, and a chandelier trocar inserted for bimanual surgery.
Step 7: The implant was introduced with special silicone-tipped forceps through the sclerotomy, and the sclerotomy temporarily closed by the assistant by pulling on the 6/0 vicryl sutures to reduce intravitreal turbulence and fluid leakage.
Step 8: The tip of the forceps holding the implant was passed through the retinotomy, and the implant was docked 1mm from the center of the fovea (primate)/central retina (feline). The larger sclerotomy for implant insertion was completely closed with 6/0 vicryl.
Step 9: A bubble of perfluorocarbon liquid (PFCL) was then injected over the retina to stabilize the implant positioned under the retina and flatten the macula.
Step 10: A laser photocoagulation at power 100-200mW was applied around the retinotomy, and PFCL removed during an air-fluid exchange.
Step 11: The macula (primate) and central retina (feline) were checked to ensure the absence of subretinal fluid. In the non-human primate study intraoperative OCT (iVue, Optovue, Fremont, CA, USA) was performed to confirm the absence of fluid under the macula.
Step 12: Either intraocular sulfhexafluoride gas or 1000 centistokes silicone oil was injected into the vitreous cavity. Subconjunctival kenalog-40 was injected, and the three sclerotomies closed with 8/0 vicryl.

During the study, a number of surgery-related adverse events were noted. The following modifications to the surgery protocol were made during the studies. The retinotomy size revised to a minimum two times the length of the optic disc to avoid iatrogenic retinal tear (Step 5). A change of incision entry to at least 3mm from the limbus (Step 6) was amended on the surgery protocol to minimize the risk of postoperative retinal detachment complications.

During creation of the subretinal bleb at the posterior pole (Step 3), the surgeon would consequently limit the intraocular subretinal fluid injection pressure to 20 psi to prevent the risk of perforating the fovea and/or macular hole development.

An overview of the key surgical PRIMA implantation steps in primate is shown in S1 Video.

## Results

### Anatomical safety and stability of the implanted PRIMA

#### Feline

All PRIMA implants were delivered below the central retina within the subretinal space in 11 feline eyes (S1 Fig), with the PRIMA microchip electrodes in contact with the retina. Immediately after the subretinal implantation, the retina was flat on top of the implants with no signs of intraretinal or subretinal fluid. There were no intraoperative complications during the follow-up period that ranged from 43–106 days.

#### Primate

Eleven primate eyes were implanted with the 1.5mm implant at the central macula (S2 Fig). In two other eyes, the retina could not be detached to allow implant delivery. There were no adverse events in these two primates at 6 weeks to 12 months follow-up period.

Four primate eyes were implanted with the 2mm implant at the central macula. (S3 Fig).

**Fig 4:**
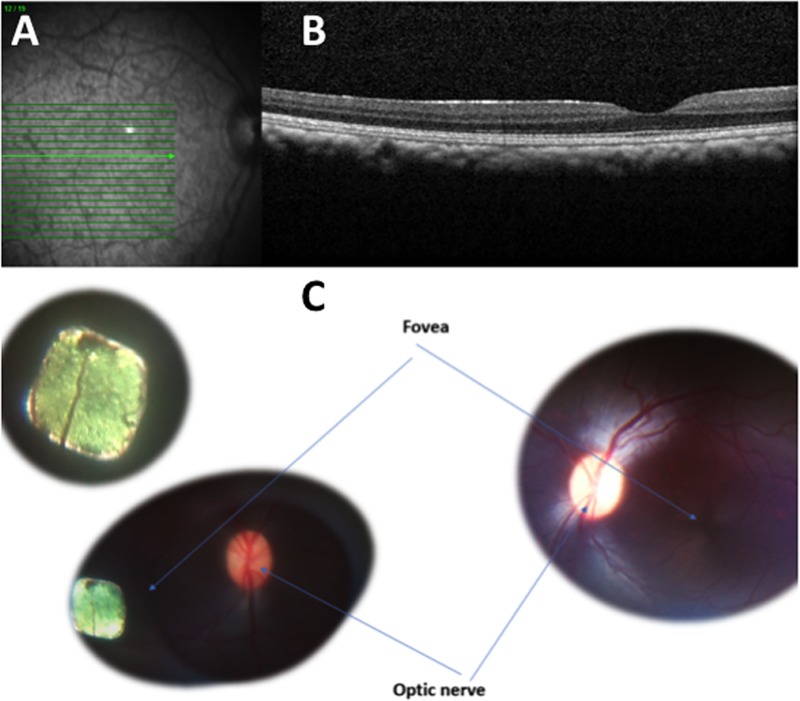
Retinal imaging of a subretinal 1.5mm PRIMA chip implantation in Primate eye before and immediately after surgery. A: Preoperative fundus photograph of macula in Macaca fascicularis; B: Preoperative optical coherence scan of macula; C: Fundus photograph of right eye 1.5mm subretinal implant with comparative left eye.

The average surgical duration for implantation of the 2mm implant in Primate eyes was 1.6 hours (range 1.5 to 2 hours) and was equivalent to the average implantation time observed for the implantation of 1.5mm implants in the study (range 2 to 3 hours including intraoperative OCT imaging, Fig 4).

**Fig 5:**
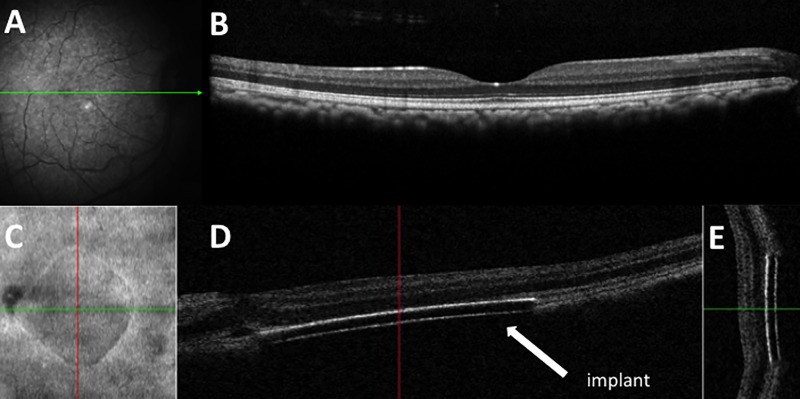
Retinal imaging of a subretinal 2mm PRIMA chip implantation in Primate eye before and immediately after surgery. A: Preoperative fundus photograph of macula in Macaca fascicularis; B: Preoperative optical coherence scan of macula; C: Enface image of the 2mm retinal implant; D: Horizontal OCT scan showing subretinal location of implant; E: Vertical OCT scan showing subretinal location of implant.

Using the 2mm implant, one subject was implanted in the left eye, three in the right eye (Fig 5).

**Fig 6:**
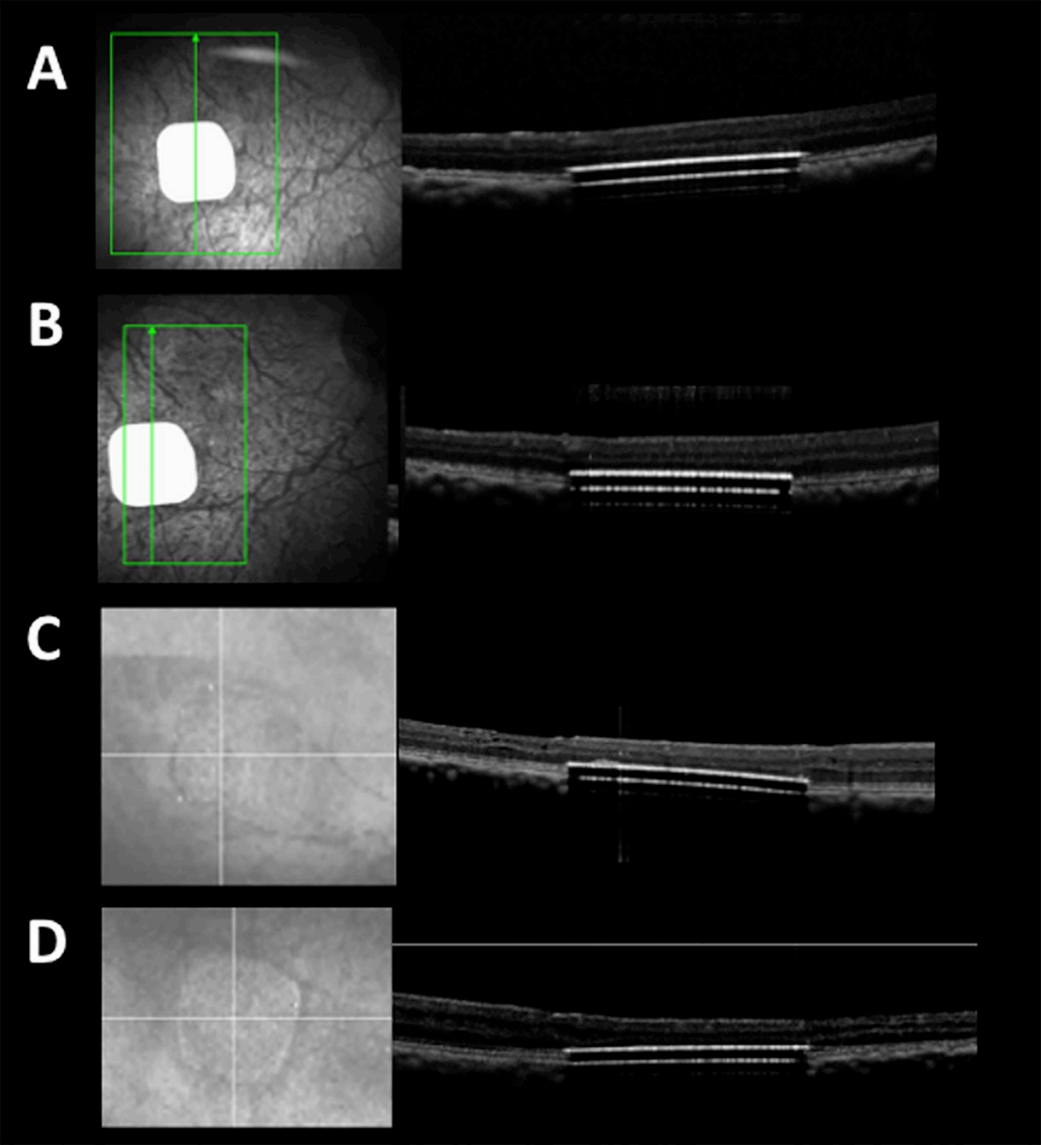
Retinal imaging of a subretinal 1.5mm PRIMA chip implantation in Primate eye at 2 weeks, 4 weeks, 8 months, and 12 months after surgery. A: 2 week postoperative optical coherence scan of macula in Macaca fascicularis; B: 4 week postoperative optical coherence scan of macula; C: 8 month postoperative optical coherence scan of macula; D: 12 month postoperative optical coherence scan of macula showing implant stability.

In addition, it has been verified that the implant was well supported by silicone oil after 2, 4 weeks, and the implant remained stable in the eye up to 1 year following implantation (Fig 6).

Immediately after the subretinal implantation, the retina was flat on top of the implants with no signs of intraretinal or subretinal fluid. In order to confirm that the surgical procedure was appropriate for the implantation of PRIMA implant in primates, the acceptance criteria listed below were defined in the protocol. (1) The implant does not break in the surgical procedure: No implant breakage occurred during all implantations, and any implant lost in the flux; (2) The implant is delivered subretinally under the fovea with a tolerance of one implant width (3) The implant did not migrate from its original position by more than one implant width: Follow up retinal and OCT images show that no implant migration occurred. (4) There was no significant retinal injury or eye damage beyond the mild AEs caused by the implantation surgery: there was no unexpected surgical complication above the standard risks of vitrectomy surgery.

In this study, all 1.5 and 2mm implants have been delivered at the target location with an off-centering of less than one implant width: either under the fovea or in the parafoveal area for two of the 1.5mm implants as this was the target area for a behavioral study conducted with these two primates [13]. In one eye, there was implant migration noted following the surgery. During PRIMA implantation, the surgeon noted implant breakage could occur if surgical handling was incorrect or excessive.

**Fig 7:**
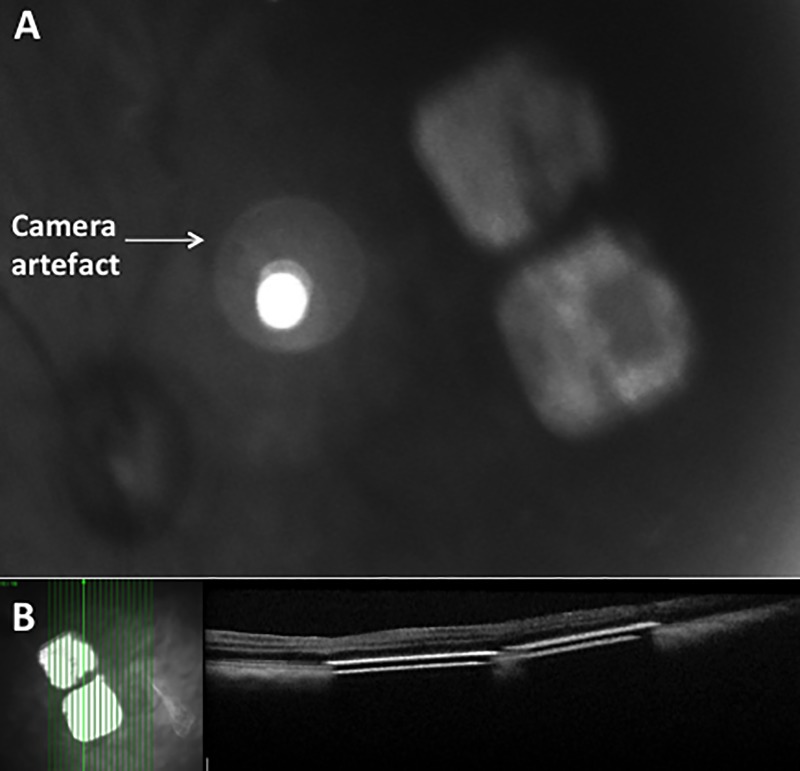
Intraoperative colour fundus photograph and OCT of 1.5mm multichip in macula of primate. A: Fundus photograph of two 1.5mm chips; B: OCT scan of 1.5mm chips placed under the macula.

In one primate eye, multi-chip 1.5mm arrays were delivered subretinally close to the fovea at a distance of respectively 0.4 and 1.5mm. The two 1.5mm implants did not touch or overlap, and there was a gap of approximately 0.2mm that remained stable (Fig 7).

### Adverse events

#### PRIMA 1.5mm implant feline surgery

In the feline study, four vitrectomy surgery-related AEs were reported in four animals at 1 and 8 days following surgery. This included two cases of corneal ulcers developing later into corneal scars, and two cases of cataract. These AE are associated risks of general intraocular surgery procedures, and were not PRIMA device-related. There were no additional AE during the follow-up period that ranged from 43–106 days.

#### PRIMA 2mm implant primate surgery

There was one case of a mild vitreous cavity hemorrhage that resolved with no complication. There was retinal tear near the retinotomy site in one animal but the retina remained attached. In one case, there was a minor area of submacular hemorrhage, with minimum hemorrhage lying on the implant surface. There was no significant postoperative inflammation, and no case of retinal detachment. A summary of non-device-related, surgical AEs is shown in Table 1.

**Table 1:** Summary of adverse events for 1.5mm and 2mm PRIMA surgery in primates.

Complication	Surgery timing	1.5mm PRIMA Implant	2mm PRIMA Implant	Postoperative, Final Ocular Status
Macular hole and retinal detachment	During subretinal injection	1	0	Minor foveal scar with attached retina
Minor submacular haemorrhage	Noted after chip insertion	0	1	Normal
Retinal tear at retinotomy with subretinal PFCL†	During PFCL injection	1	0	Retinal tear with subretinal PFCL† Retina attached
Retinal tear at retinotomy	During retinotomy creation	1	1	Retina attached
Minor vitreous cavity haemorrhage	Postoperative	0	1	Normal
Postoperative Retinal detachment	Postoperative due to entry site break	1	0	Retinal detachment treated with silicone oil. Retina attached after secondary oil removal.
Retinotomy edge tear	During creation of retinotomy	1	1	Normal
Non-surgery related retinal detachment	Postoperative after sequential oil removal	1	0	Retina attached
No implantation of PRIMA	Not possible to surgically detach the central retina/macula	2	0	Normal

† PFCL, perfluorocarbon liquid

#### PRIMA 1.5mm implant primate urgery

**Fig 8:**
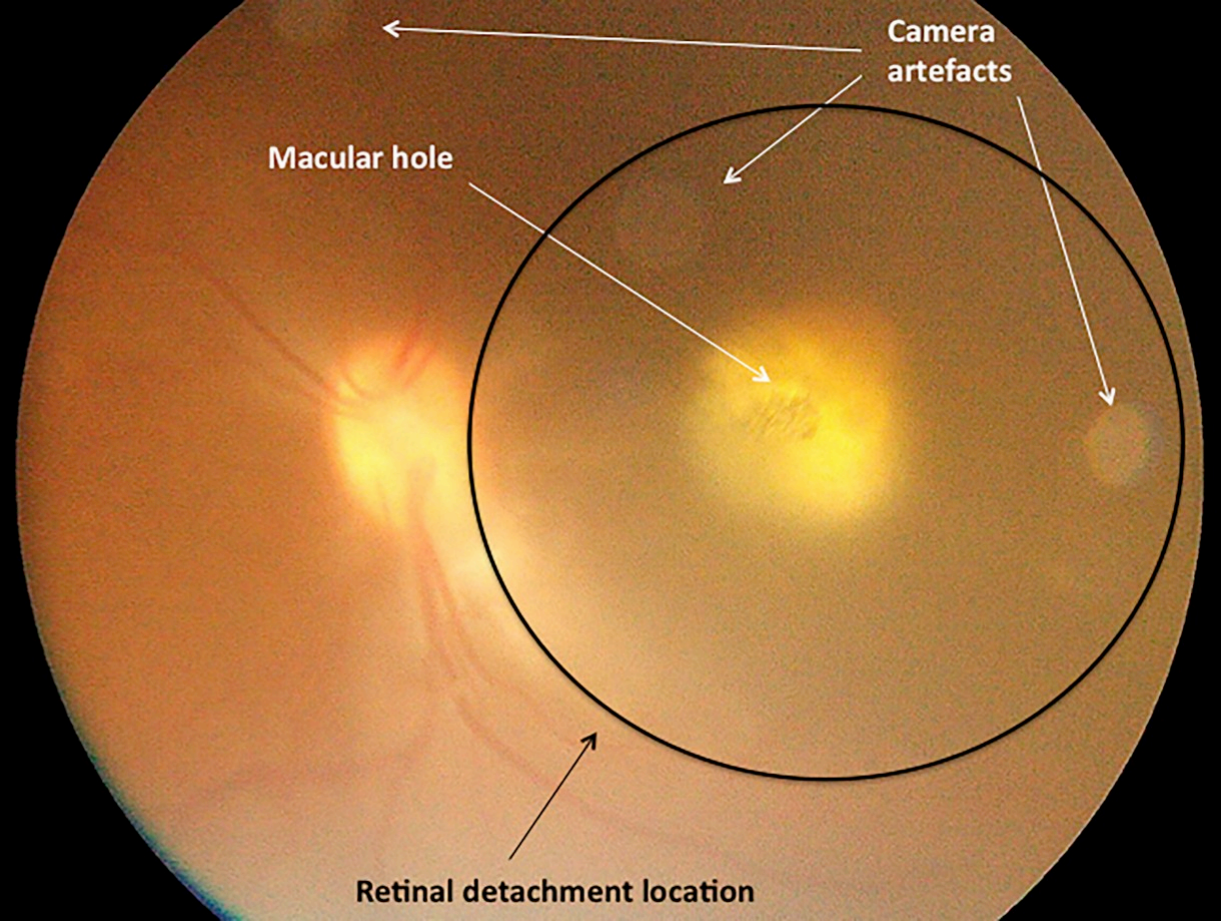
Intraoperative colour fundus photograph in macula of primate. Subfoveal 1.5mm chip placement with overlying macular hole and localised retinal detachment at macula.

A summary of non-device-related, surgical AEs is shown in Table 1. There was one case of an intraoperative macular hole associated with a retinal detachment (Fig 8).

**Fig 9:**
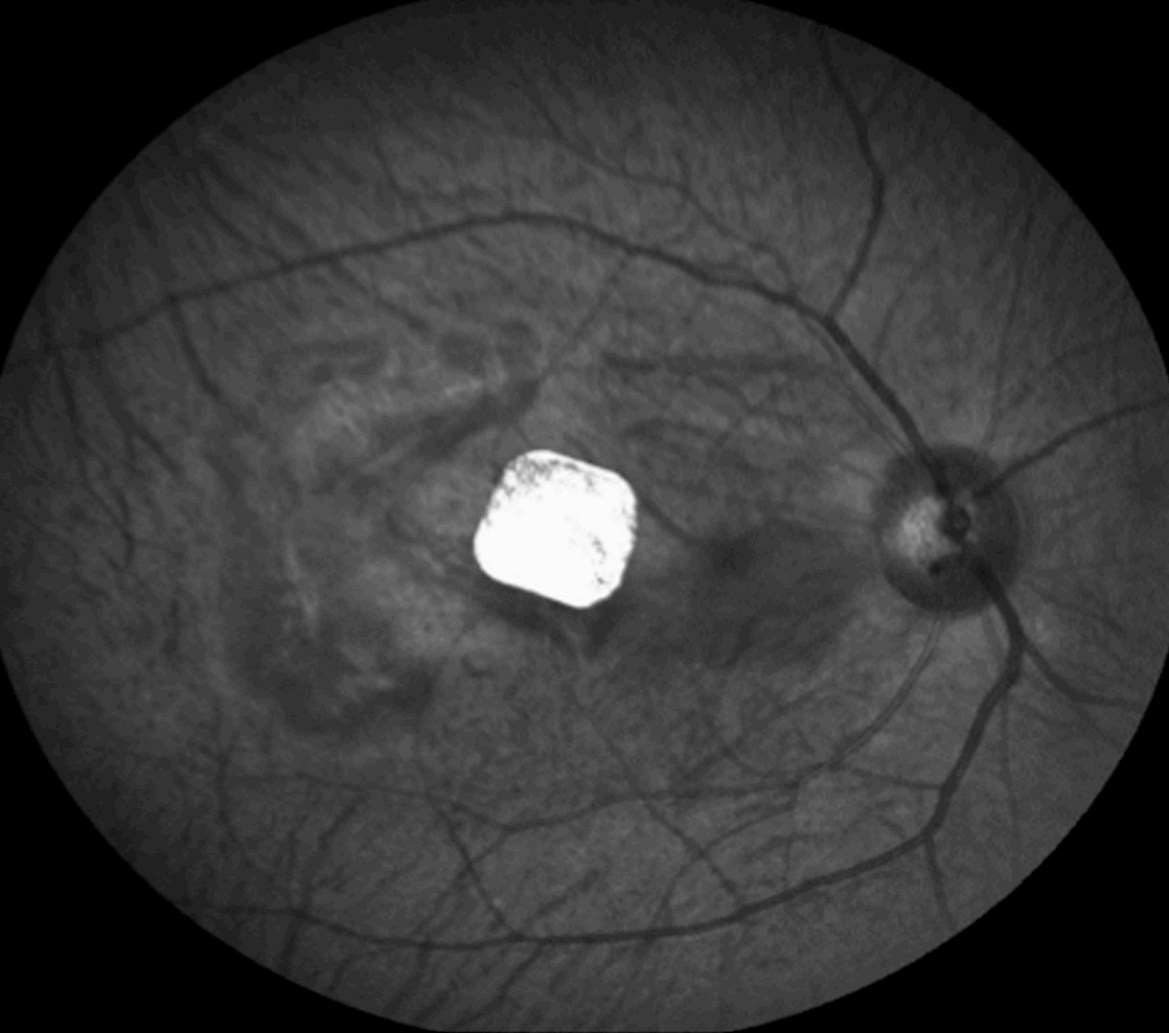
Red-free fundus photograph of in Macaca fascicularis. The 1.5mm implant remains in the central macula at 2 weeks postoperatively.

At 6 weeks follow-up, there was no significant retinal scar and the retina was attached. One case of a small bubble of PFCL retained in the subretinal space outside the zone of PRIMA implantation. There was no significant postoperative inflammation. In one eye, a retinal detachment was detected at the 6 week control. A revision vitrectomy surgery with silicone oil was performed that successfully treated this complication. In one eye, an iatrogenic tear was made at the edge of the retinotomy during implant delivery, but the implant remained centered at the fovea postoperatively (Fig 9).

After the end of 6-week follow-up period, as this study was terminated, the silicone oil was removed. Subject 1 was examined 7 days following the silicone oil removal and 17 days following oil removal surgery. At the final visit, a localised retinal detachment was detected in the posterior pole. An iatrogenic retinal tear occurred during the silicone oil removal surgery and resulted in this rhegmatogenous retinal detachment. The implant was still under the retina and close to the fovea but was not attached by the retina. A retinal detachment is a recognized complication following vitrectomy surgery and this was designated as a SAE. Three eyes were followed up for 2 years with no additional AE reported.

## Discussion

We report subretinal implantation of 1.5mm and 2mm PRIMA implants in central retina of cat eyes and central macula of Macaca fascicularis primate eyes. This study demonstrates that the surgical procedure is appropriate for both 1.5mm and 2mm PRIMA retinal implants, with slight adjustments of sclerotomy location, and the retinotomy size due to the additional 0.5mm in size.

The main purpose of this study was to assess the surgical implantation technique of two sizes of PRIMA wireless photovoltaic microchip implants in different animal models that have similar architecture to the human retina. During the studies, the surgery protocol was amended following any procedure-related AEs, and thereafter, there were no additional surgery-related AEs observed. Surgical implantation endpoints were reached in all cases. The chips were placed within the subretinal space with a single case of postoperative retinal implant migration away from the primary implantation site. The retinal implants remained stable under silicone oil in all cases with no cases of significant ocular inflammation.

We observed no device-related AE in the feline study. In the primate study, there were several minor AEs that resolved without complications at 6 weeks. One SAE of retinal detachment developed after secondary vitrectomy with silicone oil removal, but this SAE was not device-related, not primary procedure-related, nor related to the presence of the implant in the eye. A second SAE of retinal detachment occurred at the 6-week control in a different primate’s eye, and this was not related to silicone oil removal. A retinal detachment is a recognized risk in vitreoretinal surgery, and retinal detachment in similar subretinal surgery models occurs at a rate of 6 to 9% [14]. In the event of a retinal detachment, a revision vitrectomy surgery may involve either intraocular gas tamponade or silicone oil re-injection. The root cause of this initial SAE has been determined and effective counter measures are implemented to minimize future occurrence of the event. Close follow-up of patients would be instructed after silicone oil removal to detect early signs of a retinal detachment. The second SAE retinal detachment occurred in an eye where the sclerotomy incision was made at 3mm from the limbus.

Early removal of silicone oil will be planned for patients in a clinical trial as per this protocol. Silicone oil will only be intended for use as a short-term tamponade in human trials, and will be removed as soon as the retinotomy is closed with formation of a chorioretinal adhesion. The final aim of the procedure in humans is to have a subretinal chip without silicone oil. The refractive changes made by silicone oil make focus of stimulating infrared beam out of range. Silicone oil will therefore be used as a short-term tamponade and removed as soon as the retinotomy was closed.

Further analysis of the foveal perforation/macular hole AE during the study led to additional refinements of the surgery protocol. In one case, an intraoperative foveal perforation occurred at injection pressure greater than 20psi. For the remaining cases, we did not encounter any cases of foveal perforation at an injection pressure of 20psi or less [15]. In the one case of macular hole observed during chip implantation, the PRIMA microchip was implanted in the subretinal space.

In previous studies of human subretinal delivery of a retinal implant, there were no significant complications reported [9, 10]. In other in vivo subretinal interventional safety studies, there have been no significant surgical complications reported during subretinal injection delivery [16–18]. A recent in vivo safety study of subretinal gene therapy reported choroidal haemorrhage, macular hole, retinal haemorrhage, and retinal tear [19].

Considering the eye size, 18 to 20mm in the study primates versus 24mm on average in humans, a 1.5mm implant in a primate eye is equivalent to a 2mm implant in human. The study implants are otherwise equivalent to the implants intended to be implanted in human patients.

## Conclusions

This study demonstrated that both 1.5mm and 2mm PRIMA implants can be implanted safely into the subretinal space of the macula/central retina in the eye of cats and primates. A multi-chip subretinal implantation is also feasible. Based on the results of our study, modifications of the implantation surgery procedure were adopted to minimize and prevent complications. As the study models are very close to the human eye, it can be concluded that the surgical procedure is appropriate for implantation in human. The next studies of PRIMA subretinal implantation are in progress for human feasibility clinical trials.

## Supporting information

S1 Fig Fundus photographs and optical coherence scans in feline eyes. Fundus photographs and optical coherence scans of 10 feline eyes.
(TIF)

S2 Fig Fundus imaging and OCT scanning of 1.5mm chip placement in primate eyes. Fundus photographs and optical coherence scans of the implanted macula of Macaca fascicularis.
(TIF)

S3 Fig Fundus imaging and OCT scanning of 2mm chip placement in primate eyes. Fundus photographs and optical coherence scans of the implanted macula of Macaca fascicularis.
(TIF)

S1 Video Video clip of PRIMA implantation under macula of Macaca fascicularis. (MP4)
