# Supplementary figures and images for: PRIMA subretinal wireless photovoltaic microchip implantation in non-human primate and feline models
Source: PLoS One. 2020 Apr 8;15(4):e0230713. doi: 10.1371/journal.pone.0230713 (PMC7141693; doi:10.1371/journal.pone.0230713)

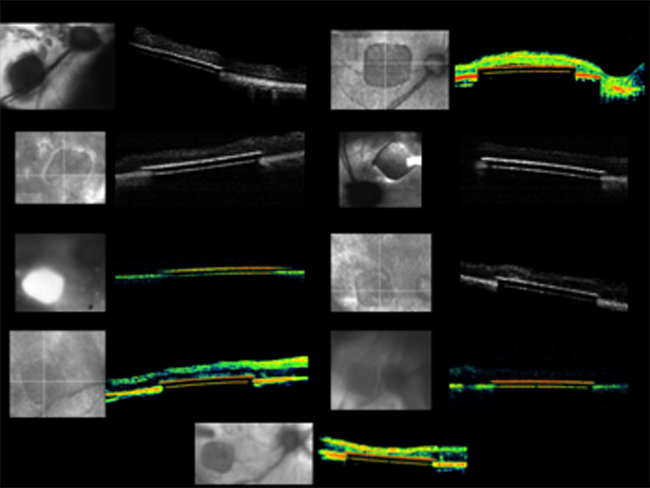

Supplement: S1 Fig — Fundus photographs and optical coherence scans of 10 feline eyes. (TIF) [file pone.0230713.s001.tif]

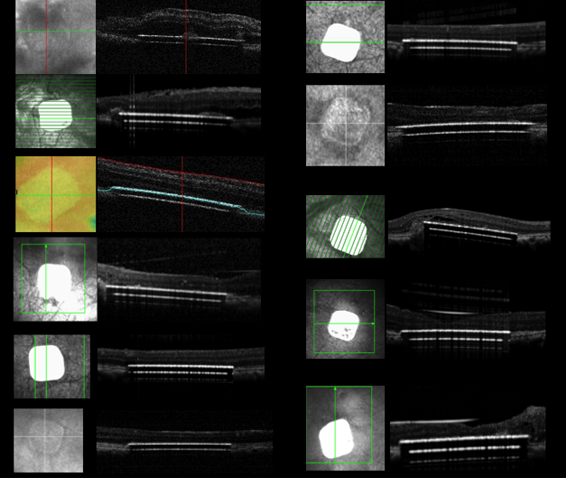

Supplement: S2 Fig — Fundus photographs and optical coherence scans of the implanted macula of Macaca fascicularis. (TIF) [file pone.0230713.s002.tif]

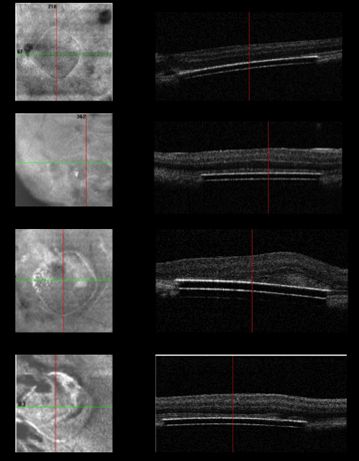

Supplement: S3 Fig — Fundus photographs and optical coherence scans of the implanted macula of Macaca fascicularis. (TIF) [file pone.0230713.s003.tif]
